# Magnetic resonance arthrography of the shoulder: a painful procedure?

**DOI:** 10.1590/0100-3984.2016.0226

**Published:** 2018

**Authors:** Paulo César Xavier do Nascimento, André Maltez Amaral, João Ricardo Maltez de Almeida

**Affiliations:** 1 Biomedical Professional, Graduate Student in Bioimaging at the Escola Bahiana de Medicina e Saúde Pública, Salvador, BA, Brazil.; 2 MD, Radiologist at the Clínica de Assistência à Mulher - CAM, Salvador, BA, Brazil.; 3 PhD, Radiologist at the Clínica de Assistência à Mulher - CAM, Salvador, BA, Brazil.

**Keywords:** Magnetic resonance imaging, Arthrography, Shoulder, Pain, Visual analog scale, Ressonância magnética, Artrografia, Ombro, Dor, Escala visual analógica

## Abstract

**Objective:**

To compare the pain expected to that effectively caused by magnetic resonance arthrography of the shoulder and, secondarily, to describe a simplified approach to the technique for articular access.

**Materials and Methods:**

We prospectively evaluated 40 participants who used a visual analog scale and a simplified categorical scale to indicate the level of pain expected and that experienced after the procedure, comparing the two with the Wilcoxon matched-pairs test. We also determined gender-related differences in pain conditions using the Mann-Whitney U test. In addition, we described a modified technique involving radiographic localization and the use of standard puncture needles for articular access.

**Results:**

Analysis of the visual analog scales showed that the pain experienced was less than had been expected, with median scores of 1.75 and 3.75, respectively (*p* < 0.001). The level of pain expected was higher among women than among men, with median scores of 8.0 and 3.0, respectively (*p* = 0.014), as was the level of pain experienced, with median scores of 3.0 and 1.5, respectively (*p* = 0.139). The overall categorical evaluation corroborated that difference (*p* = 0.03). Articular access with the modified technique was successful in all patients.

**Conclusion:**

Magnetic resonance arthrography of the shoulder is less painful than patients expect. In addition, digital radiographic guidance combined with the use of standard puncture needles appears to improve the efficiency of the method.

## Abstract (pt)

**Objetivo:**

Comparar a expectativa de dor com a efetivamente produzida em procedimentos de artrorressonância do ombro e, secundariamente, descrever abordagem simplificada da técnica de acesso articular.

**Materiais e Métodos:**

Avaliamos, prospectivamente, 40 participantes, que assinalaram a sua expectativa álgica e a dor pós-procedimento em escalas visual analógica e categórica simplificada, que foram comparadas por meio de teste pareado de Wilcoxon. Também determinamos a diferença do quadro doloroso por gênero, com aplicação do teste U de Mann-Whitney. Descrevemos, ainda, técnica de localização radiográfica e emprego de agulhas simples de punção para acesso articular.

**Resultados:**

A análise das escalas visuais analógicas demonstrou que a dor experimentada foi inferior à esperada (medianas de 1,75 e 3,75, respectivamente; *p* < 0,001). As mulheres tinham expectativa de dor superior aos homens (medianas de 8,0 e 3,0; *p* = 0,014), assim como maior dor experimentada (medianas de 3,0 e 1,5; *p* = 0,139), respectivamente. Avaliação categórica global corroborou tal tendência (*p* = 0,03). O acesso articular, empregando técnica adaptada, foi obtido com sucesso em todos os pacientes.

**Conclusão:**

A artrorressonância do ombro é menos dolorosa do que se imagina. Adicionalmente, a orientação radiográfica digital aliada ao emprego de agulhas simples de punção aumentam a eficiência do método.

## INTRODUCTION

Shoulder pain is one of the most common complaints related to the musculoskeletal system^(1,2)^. Similar signs and symptoms can be produced by injuries to various structures and can lead to significant functional disability when not properly diagnosed^(3)^. Therefore, it is fundamental that the different diagnostic modalities be indicated correctly. One such modality is magnetic resonance arthrography, better known as MR arthrography or MRA, which plays a prominent role because of its high accuracy, particularly in the evaluation of glenohumeral instability, due to its superior detailing of ligamentous, cartilaginous, and labral structures^(4,5)^.

The MRA examination is invasive in nature, because the joint space is accessed by guided puncture, followed by injection of a contrast agent. Therefore, it is often characterized as intensely painful, not only by uninformed patients but also by healthcare professionals who are themselves uneducated regarding the technique. However, as in any subjective experience, there is a relevant emotional component, which makes it extremely difficult to measure pain by quantitative methods^(6-8)^. In addition, there are inherent technical variations, ranging from the type of needle used-usually the type used in lumbar puncture^(1,9,10)^-to the dilution of the contrast agent^(11)^ and the approach to accessing the glenohumeral joint^(9,12,13)^.

The main objective of this study was to compare the expectation of pain related to shoulder MRA (level of pain expected) with the pain actually produced by the procedure (level of pain experienced), by applying, at different time points, a visual analog scale (VAS) and a simplified numerical categorical scale. A secondary objective was to describe an adaptation of the technique of localization and anterior puncture through the rotator interval^(13-16)^, which simplifies the procedure and reduces its cost, thus increasing its efficiency.

## MATERIALS AND METHODS

Between July 2015 and March 2016, patients undergoing shoulder MRA in the Bioimaging Department of the Clínica de Assistência à Mulher - Grupo CAM, in the city of Salvador, Brazil, were invited to participate in the study. Patients who had previously undergone MRA of any joint were excluded, as were those who required sedation (no such cases being identified during the sample selection). The final sample comprised 40 patients, all of whom had been referred by orthopedists or other specialists, none of whom were affiliated with the study. The study was approved by the Research Ethics Committee of the Bahiana School of Medicine and Public Health, also in the city of Salvador (Ruling No. 1,195,717). All participating patients gave written informed consent.

**Figure 1:**
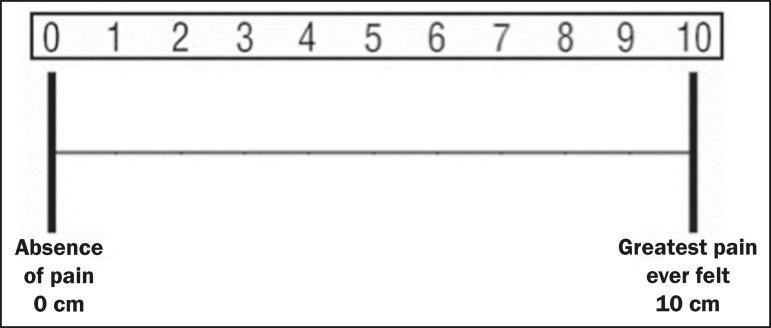
The VAS, arranged horizontally, the leftmost point corresponding to the absence of pain and the rightmost point corresponding to the greatest pain ever felt.

Prior to the procedure, a trained nurse informed the participant of the basic steps to be followed, with specific reference to fine-needle joint puncture and contrast agent injection, as well as the subsequent examination in the magnetic resonance imaging (MRI) scanner. The participant then indicated the level of pain expected, on a standardized VAS, consisting of a straight 10.0-cm line, the leftmost point (0.0 cm) corresponding to the absence of pain and the rightmost point (10.0 cm) corresponding to the greatest pain ever felt (Figure 1). The participants also employed a categorical scale to indicate the level of pain. For simplification and reiteration of the sensorial expectation, the categorical scale comprised five levels: 1, no pain; 2, mild pain; 3, moderate pain; 4, severe pain; and 5, maximum pain. After the examination, the VAS and the categorical scale were reapplied in order to assess the actual level of pain experienced. The participants completed both scales in a private room, with no supervision by the clinic staff. At approximately 4 h after the end of the examination, the participants were contacted by text message or telephone call, in which they were asked to classify their current level of pain from 1 to 5 according to the categorical scale. Nine of the participants were lost to that follow-up, and three of those nine did not indicate the level of pain on the categorical scale immediately after the examination (all participants performed the VAS marking). Therefore, in order to compare the categorical scales between groups of different sizes, we constructed a normalized index-a pain index-by dividing the sum of the categories of pain at each time point and dividing that by the maximum possible score, which was calculated by multiplying it by the number of participants by 5 (maximum pain category).

### Examination protocol

**Figure 2:**
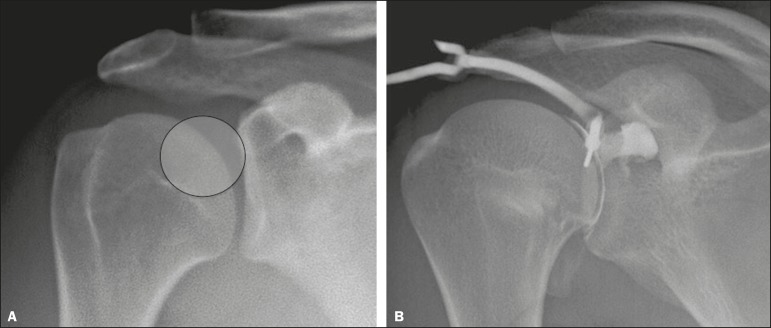
Digital radiograph of the shoulder (**A**) showing the ideal place for joint puncture (circle), over the upper third of the humeral head, near the glenohumeral joint. Injection of the iodinated contrast medium (**B**), via a dual-lumen port, confirming access to the joint.

All procedures were performed by one of two radiologists trained in the bioimaging sector (examiners 1 and 2), who used a puncture technique directed to the rotator interval, in the vicinity of the joint cleft, by means of an approach previously described^(13-16)^. That technique was adapted for the use of conventional digital radiography, rather than fluoroscopy, as a guide for determining the puncture site. The participants were placed in the supine position, with slight external rotation of the shoulder, and those who reported pain in that position were allowed to remain in neutral rotation^(15)^. After palpation of the tip of the coracoid process and estimation of the location of the joint cleft, the radiologist applied a marker (metal clip) on the shoulder as a reference for the site and the puncture point was marked with a pen (Figure 2A). The radiologist diluted a gadolinium-based contrast agent-0.1 mL of meglumine gadoterate (Dotarem; Guerbet, Paris, France)-in a mixture of 10.0 mL of 1% lidocaine hydrochloride, without a vasoconstrictor (Xylestesin, Cristália, São Paulo, Brazil), and 10.0 mL of sterile saline solution. A slight impression was then made in the skin, over the designated location, by pressing with a ballpoint pen tip, with the pen retracted (because an ink mark would be erased when the skin was cleaned). After rigorous asepsis, local anesthesia was achieved with an intradermal injection of approximately 3.0 mL of 1% lidocaine hydrochloride, without a vasoconstrictor. At that time, with the needle *in loco*, conventional radiography was performed to confirm the proper positioning. The joint was then accessed by introducing a 21 G, 0.8 × 40 mm disposable needle, through which 2.0-3.0 mL of iodinated contrast medium containing organically bound iodine (Henetix; Guerbet) were injected for confirmation of articular access, with a dual-lumen port (Polifix; B. Braun, Rio de Janeiro, Brazil), as depicted in Figure 2B. If the distribution of the iodinated contrast agent was as expected, its channel was closed and, through the second channel, 10.0-13.0 mL of the gadolinium-based contrast agent were introduced.

After the contrast agents had been injected into the joint, the participant was placed in an MRI scanner (Signa HDxt; General Electric, Waukesha, WI, USA). Multiplanar images of the shoulder were acquired in a < 40-min protocol that included an abduction and external rotation sequence as the final acquisition. For all of the participants in the sample, the MRA examinations were considered to be of satisfactory quality.

### Statistical analysis

We calculated a sample size of 35 participants for a nonparametric Wilcoxon matched-pairs test, using an estimated effect size of 0.50, a desired power of 0.80, and a statistical significance of 0.05. We described the data related to the sample composition by measures of central tendency and variability, using parametric or nonparametric methods, according to their distribution, as determined by the Shapiro-Wilk test. The pain scales used (the VAS and the categorical scale) were treated in an ordinal manner and were compared by the Wilcoxon matched-pairs test. We also used the Mann-Whitney U test in order to compare the genders in terms of pain, on the basis of the difference between the level of pain expected and that experienced, as well as to compare the two examiners in terms of the results obtained.

The sample size was calculated using the G*Power software, version 3.9.1.2 (Heinrich-Heine University, Düsseldorf, Germany), and the other analyses were performed with the IBM SPSS Statistics software package, version 19.0 (IBM Corporation, Armonk, NY, USA). All tests were two-tailed, and the level of statistical significance adopted was 5% (*p* < 0.05).

## RESULTS

Of the 40 patients who participated in the study, 29 (72.5%) were male and 11 (27.5%) were female. The overall mean age was 30.7 years (standard deviation [SD] = 9.92 years). Among the participants, the mean weight was 78.28 kg (SD = 13.62 kg) and the mean height was 1.73 m (SD = 0.083 m). Immediately before the MRA, 25 (62.5%) of the participants reported feeling no shoulder pain, which was reported by the remaining 15 (37.5%). Only 5 of the participants (12.5%) had previously undergone surgery on the shoulder examined.

**Figure 3:**
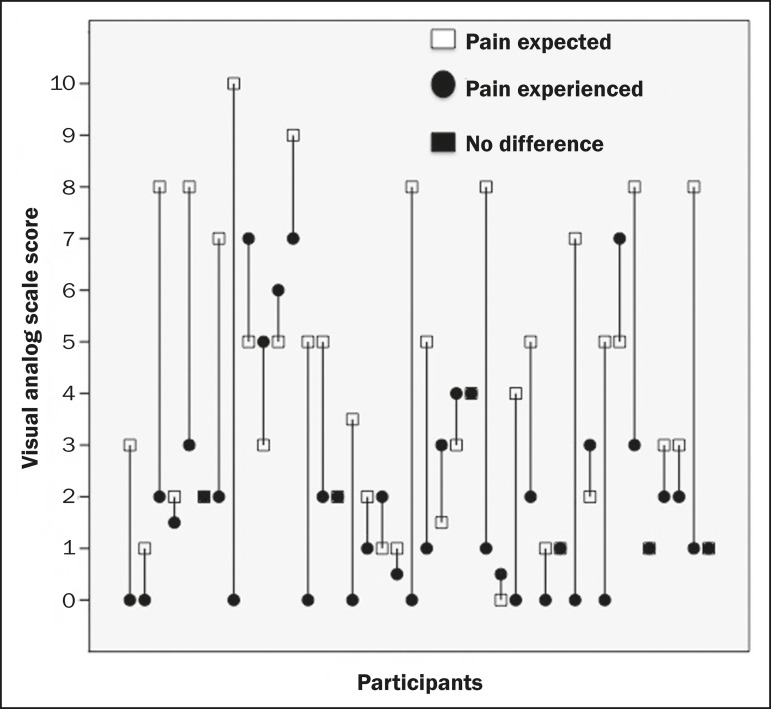
Graphic showing the pain expected and that experienced immediately after the procedure, by participant, based on the VAS markings.

**Figure 4:**
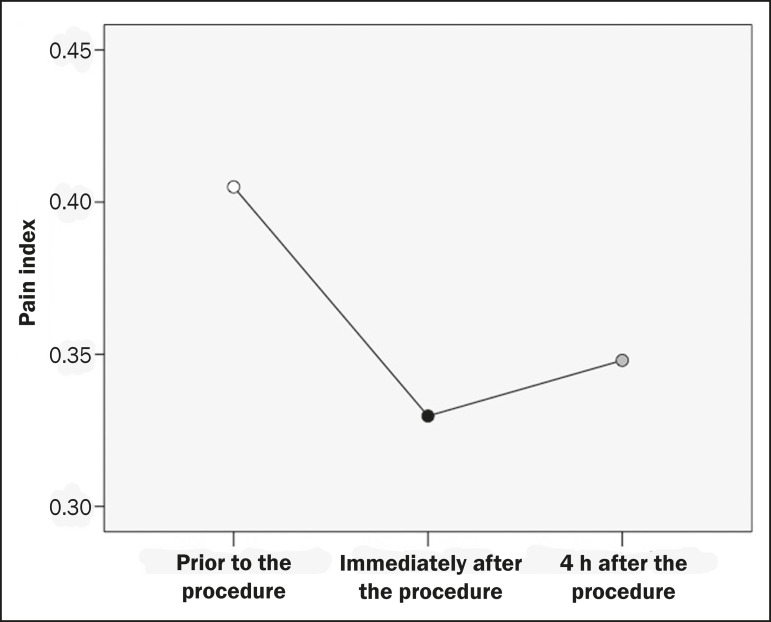
Line graph showing a reduction in the pain index immediately after the procedure, with a slight upward trend at 4 h thereafter.

Analysis of the VAS results showed that the participants had an expectation of pain, with a median score of 3.75 (interquartile range [IQR] = 4.50), considerably higher than the median score for the pain actually experienced, which was 1.75 (IQR = 2.88). As can be seen in Figure 3, the reported level of pain experienced was lower than expected in 25 participants (62.5%), higher than expected in 9 (22.5%), and exactly as expected in 6 (15%). The Wilcoxon matched-pairs test showed that the pain expected was significantly worse than the actual pain experienced (*p* < 0.001). The evaluation of the scores reported on the numerical categorized scales revealed the same tendency (Table 1). Of the 37 participants who completed the categorical scale immediately after the examination, none of the participants reported severe (category 4) or maximum (category 5) pain, and only 3 (8.1%) reported moderate (category 3) pain, whereas 34 (91.9%) reported no pain or mild pain (category 1 or 2). The difference between the categorical scores reported before the procedure and those reported immediately after the procedure was significant (*p* = 0.03). Among the 31 participants contacted subsequently, the level of pain reported at 4 h after the procedure did not differ significantly from that reported immediately after the procedure (*p* = 0.519). However, the graphic interpretation of the pain indices (Figure 4) revealed a slight trend toward an increase in the level of pain reported at 4 h after the procedure.

**Table 1:** Number of participants per category of pain at different time points, with summation of the scores and calculation of the pain index.

	Prior to the procedure			Immediately after the procedure			4 h after the procedure	
	(n = 37)			(n = 37)			(n = 31)	
Categorical pain scale score	N	(%)*		N	(%)*		N	(%)*
1 (no pain)	12	(32.4)		16	(43.2)		15	(48.4)
2 (mild pain)	16	(43.2)		18	(48.6)		10	(32.3)
3 (moderate pain)	6	(16.2)		3	(8,1)		5	(16.1)
4 (severe pain)	2	(5.4)		-	-		1	(3.2)
5 (maximum pain)	1	(2.7)		-	-		-	-
Total	75			61			54	
Pain index^†^, median	0.41			0.33			0.35	

* Due to rounding, percentages might not total 100%.

† Corresponds to the sum of the categorical pain scale scores, for a given participant, at the different time points, divided by the maximum possible score: [sum/(n*5)].

**Figure 5:**
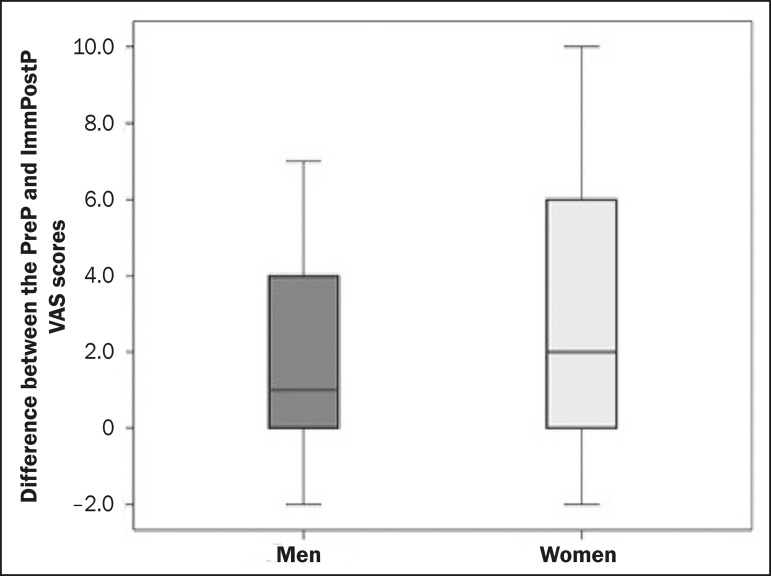
Box plot of the difference between the VAS scores for the pain expected (PreP VAS scores) and for the pain experienced immediately after the examination (ImmPostP VAS scores), by gender, showing no significant difference between the men and women in terms of the median score.

The level of pain expected was considerably higher among the women than among the men, the median scores being 8.00 (IQR = 4.00) and 3.00 (IQR = 3.25), respectively (*p* = 0.014). Although the same trend was observed for the level of pain effectively experienced-the median scores for women and men being 3.00 (IQR = 6.00) and 1.50 (IQR = 2.00), respectively-the difference was not statistically significant (*p* = 0.139). The pain expected was significantly worse than the pain experienced, even when the genders were evaluated separately (*p* = 0.003 for males and *p* = 0.049 for females). The median of the simple subtraction between the VAS score for the pain expected and that for the pain experienced was similar between the genders (Figure 5), with no relevant difference demonstrable by the Mann-Whitney U test (*p* < 0.563). Similarly, a comparison between the participants who reported pain before the procedure and those who did not showed that the difference between the two groups was not significant (*p* = 0.705).

Examiner 1 performed 16 procedures, and examiner 2 performed 24 procedures. The analysis of the VAS scores revealed no significant differences between the two examiners, the median VAS score for the pain expected being 5.00 (IQR = 4.50) for the MRAs performed by examiner 1, compared with 3.00 (IQR = 5.25) for those performed by examiner 2 (*p* = 0.503), whereas the median VAS score for the pain actually experienced was 1.50 (IQR = 2.75) and 1.75 (IQR = 2.63) for the MRAs performed by examiner 1 and examiner 2, respectively (*p* = 0.855). The difference between the pain expected and that experienced, when evaluated by examiner, was also not significant (*p* = 0.729).

In all of the examinations, the application of digital radiography, without the use of fluoroscopy, together with the use of a standard puncture needle, allowed easy access to the joint. In our sample, the examiners made no errors during the procedure; that is, there were no cases in which the needle had to be repositioned or the patient had to recalled.

## DISCUSSION

Recent studies in the radiology literature have emphasized the role of MRI in the evaluation of the musculoskeletal system^(17-21)^. In the present study, we compared the pain expected by patients scheduled to undergo shoulder MRA with the pain effectively experienced after the procedure, applying a VAS and a categorical scale. Our results indicate that the level of pain expected is considerably higher than the pain effectively experienced. That difference was observed in both genders, independently. In addition, we have described an adaptation of the anterior articular access technique through the rotator interval^(13-16)^, using digital radiography to locate the puncture portal, as well as the use of a standard needle rather than spinal needles.

For examination of the shoulder, conventional MRI has been preferred over MRA, despite the high sensitivity and specificity of the latter^(5,22)^. Among the reasons given for that is the invasive nature of shoulder MRA, which discourages many patients from undergoing the procedure. Robbins et al.^(23)^ reported that "pain" and "needles" were among the main fears reported by patients in relation to MRA examination of various structures. However, the authors demonstrated that the pain experienced was usually less than that expected, only 6% of the participants reporting pain greater than that expected, which is in keeping with our results.

Blanchard et al.^(24)^ compared conventional MRI of the shoulder with conventional arthrography of the shoulder, in terms of the levels of anxiety and pain. They found that the number of patients describing the experience as "uncomfortable" or "extremely uncomfortable" was significantly higher among those undergoing MRI than among those undergoing arthrography. However, unlike what we observed in the present study, those authors found that the levels of anxiety and pain were significantly higher among women than among men.

In a study evaluating MRA of different joints in 1085 participants, Saupe et al.^(10)^ employed methods similar to those used in the present study. Their results showed that the level of pain reported immediately after the procedure was lower than that reported prior to the procedure. However, the authors found that the pain profile was related to the time factor, a statistically significant worsening of pain being observed at 4 h after the procedure, particularly after shoulder MRA, a tendency that was also observed in the present study, although the difference did not reach the level of statistical significance in our sample. That observation was attributed to the wearing off of the anesthetic effect, and there was progressive improvement within one week thereafter. Although those authors also found variations by age group, they did not observe relevant differences associated with the joint involved, the type of paramagnetic contrast used, and gender. The puncture point used in their study (superomedial portion of the humeral head) was similar to that employed in the present study.

In 1933, Oberholzer^(25)^ described the technique of glenohumeral joint access for arthrography, which was simplified in 1975 by Schneider et al.^(26)^, who used fluoroscopy to guide the puncture needle to the middle/lower third of the joint. That approach transfixes the anterior stabilizing structures of the shoulder and has the potential to cause local anatomical distortion and, in some cases, iatrogenic lesions^(10,12)^. Over time, new methods of joint puncture were developed, and anterior access through the rotator interval became widely accepted after the works of Berná-Serna et al.^(13)^, Dépelteau et al.^(15)^, and Redondo et al.^(16)^, all of which were published between 2004 and 2008.

In Brazil, most private diagnostic imaging clinics do not offer fluoroscopy. In the present study, we have demonstrated that the digital radiography approach to guiding joint puncture is a viable and efficient option, because it allowed easy and uneventful access in all participants. The training of the performing physician has a direct influence on the success rate, just as the success of the fluoroscopy-guided version of the approach is affected by the experience of the radiologist^(15,16)^. For example, in the study conducted by Dépelteau et al.^(15)^, needle repositioning was necessary in 6 (15%) of the 40 cases in which the procedure was performed by residents. In contrast, Redondo et al.^(16)^ reported such failures in only 2 (2.5%) of 78 cases. Although our sample was small in size, the examiners were successful in all of the procedures, without statistically relevant differences between the two examiners in terms of the level of pain reported. That could be at least partially attributable to the fact that both examiners had been trained in the same technique and instructed to follow the protocol to the letter. We found the performance of the digital radiography-guided method to be satisfactory.

In the present study, another variation from the typically recommended technique^(9,16,23)^ was the use of a standard puncture needle rather than a spinal needle. That adaptation is aimed at further improving the efficiency of the procedure, because standard needles are considerably less costly^(27)^. Admittedly, the cost of MRA is higher than is that of other methods of evaluating the shoulder, regardless of the setting in which they are employed^(28)^. In Brazil, the aggregate difficulties of obtaining financial reimbursement from health insurance plans for the material used constitute a significant obstacle, often precluding the use of MRA in private practice.

Our study has some limitations, primarily those inherent to the evaluation of an experience as subjective as pain^(29,30)^. However, we believe that we were able to mitigate those impediments, at least in part, by using two different scales (a VAS and a categorical scale) in parallel, treating them conservatively by applying nonparametric tests. In addition, the sample size calculation was performed with the primary objective in mind, which reduced the statistical power for secondary evaluations of certain characteristics inherent to the group studied. Furthermore, three participants did not use the categorical scale immediately after the examination and nine were lost to follow-up (i.e., could not be contacted at 4 h after the examination), which also reduced the statistical power. We believe that the three participants in question simply neglected to mark the categorical scale, because it was on the second page of the handout. On the VAS, two of those three participants had indicated that the level of pain after the procedure was lower than expected, whereas one had indicated that it was exactly as expected. To avoid any bias related to the expectations of the observer, we opted not to supervise the marking of the scales. Another potential limitation is that we did not draw comparisons among different articular access sites or different needles, although such comparisons were not included in the original objectives of the study.

## CONCLUSION

There have been few studies, especially in Brazil, aimed at the qualitative or semiquantitative evaluation of the pain related to MRA procedures. Therefore, the present study aims to fill that gap by demonstrating that shoulder MRA is less painful than patients typically expect. In addition, our findings indicate that fluoroscopy can be dispensed with for radiographic guidance of articular access and that shoulder MRA can be performed with a standard puncture needle, both of which help reduce the costs of the examination and increase its efficiency.
